# A Comparison of Radiologic Tumor Volume and Pathologic Tumor Volume in Renal Cell Carcinoma (RCC)

**DOI:** 10.1371/journal.pone.0122019

**Published:** 2015-03-23

**Authors:** See Min Choi, Don Kyoung Choi, Tae Heon Kim, Byong Chang Jeong, Seong Il Seo, Seong Soo Jeon, Hyun Moo Lee, Han-Yong Choi, Hwang Gyun Jeon

**Affiliations:** 1 Department of Urology, Gyeongsang National University Hospital, Gyeongsang National University School of medicine, Jinju, Korea; 2 Department of Urology, Samsung Medical Center, Sungkyunkwan University School of Medicine, Seoul, Korea; 3 Department of Urology, Samsung Medical Center, Samsung Biomedical Research Institute, Sungkyunkwan University School of Medicine, Seoul, Korea; University of Nebraska Medical Center, UNITED STATES

## Abstract

**Objective:**

To investigate the difference between preoperative radiologic tumor volume (RTV) and postoperative pathologic tumor volume (PTV) in patients who received nephrectomy for renal cell carcinoma (RCC).

**Materials and Methods:**

We reviewed 482 patients who underwent preoperative computed tomography (CT) within 4 weeks before radical or partial nephrectomy for renal cell carcinoma. RTV measured by a three dimensional rendering program was compared with PTV (π/6ⅹheightⅹlengthⅹwidth) measured in surgical specimen according to pathologic tumor size and histologic subtype. Correlation of the inter-quartile range (IQR) of the RTV and Fuhrman nuclear grade was also investigated.

**Results:**

There was a significant positive linear correlation between RTV and PTV (p<0.001, r = 0.911), and the mean RTV and mean PTV were not significantly different (79.0 vs 76.9cm^3^, p = 0.393). For pathologic tumor size (PTS) <4cm, the mean RTV was larger than the mean PTV (10.9 vs 7.1cm^3^, p<0.001). For a PTS of 4-7cm, the mean RTV was larger than the mean PTV (56.0 vs 44.7cm^3^, p<0.001). However, for a PTS ≥7cm, there was no statistical difference between RTV and PTV (p>0.05). Among patients with clear cell RCC, the mean RTV was significantly larger than the mean PTV (p = 0.042), not for non-clear cell group (p = 0.055). As the quartile of the RTV increased, the Fuhrman grade also increased (p<0.001).

**Conclusions:**

RTV was correlated with PTV and pathologic grade. RTV was larger than the PTV for a tumor size 7 cm or less or in clear cell RCC. RTV may be useful to measure tumor burden preoperatively.

## Introduction

An increasing number of small renal masses are detected with modern cross-sectional imaging [1]. Thus, the frequency of nephron sparing surgery, such as partial nephrectomy (PN), has increased. At the same time, complete resection of the renal mass and preservation of renal function is of the utmost importance. As a result, accurate measurement of a renal mass is required, and many studies have been conducted

There have been several studies comparing radiologic tumor size (RTS) and pathologic tumor size (PTS) in renal cell carcinoma (RCC) [2–4]. Although there have been slightly different findings in each study, the RTS of clear cell RCC was overestimated by 0.23 cm to 0.97 cm compared to PTS [2, 5, 6]. Recently, there have been several efforts to measure the renal tumor volume. Thiel et al. measured pathologic tumor volume (PTV) in RCC by an ellipsoid formula using three measurements from the pathologic report [7]. Aerten et al. measured radiologic tumor volume (RTV) in RCC with a medical imaging post-processing system [8]. Secil et al. calculated RTV using the conventional method of multiplication of three sizes of the tumor on computed tomography (CT) images, and they also measured RTV by using a post-processing workstation which enabled three-dimensional (3D) image processing [9]. With the advent of these methods, there have also been several studies investigating the correlation between RTV and survival in RCC patients [9, 10].

Judging from previous studies comparing RTS and PTS, the RTV is expected to overestimate the PTV. However, there have been no studies that compare RTV and PTV after renal surgery in RCC patients. Thus, we investigated the correlation between RTV via preoperative CT images and PTV in surgical specimens according to tumor size, histologic subtype and Fuhrman grade in patients who received radical or partial nephrectomy for RCC.

## Materials and Methods

### Patients

This retrospective study was approved by the Samsung Medical Center (SMC) institutional review board. IRB file number is SMC 2014-03-127-003. Records and informations of patients were anonymized and deidentified prior to analysis. The medical records and images of patients treated by radical nephrectomy (RN) or PN from March 2001 to December 2012 were reviewed. Patients whose preoperative CT images were taken within 28 days of renal surgery were included in this study. Patients who exhibited multiple, bilateral, or cystic renal masses on preoperative CT were excluded.

### CT protocol

CT scans were performed by using one of five multidetector units (LightSpeed QX/I, LightSpeed Ultra8, LightSpeed Ultra16, and LightSpeed VCT, GE Medical System, Milwaukee, Wis; Brilliance 40, Philips Medical Systems, Cleveland, Ohio). The parameters for the unenhanced and contrast-enhanced examinations included 0.625–5 mm collimation, pitch of 0.750–0.984, reconstructed section thickness of 3–5 mm, 120 kVp, and 180–240 mAs[11]. Contrast-enhanced CT scans were obtained after intravenous injection of 120 ml of nonionic contrast medium at a rate of 2.5–3.0 ml/sec by using a power injector. The kidney CT protocol included an unenhanced CT and two-phase contrast-enhanced CT in which images were obtained 30 seconds (arterial phase) and 3 minutes (venous phase) after bolus contrast material injection.

### Measurement of tumor size and volume

All CT images were evaluated by two experienced radiologists. RTS was defined as the maximal tumor diameter in preoperative CT images. Pathologic assessment were done by one experienced pathologist. PTS was defined as the maximal tumor diameter from the postoperative pathologic results.

**Fig 1:**
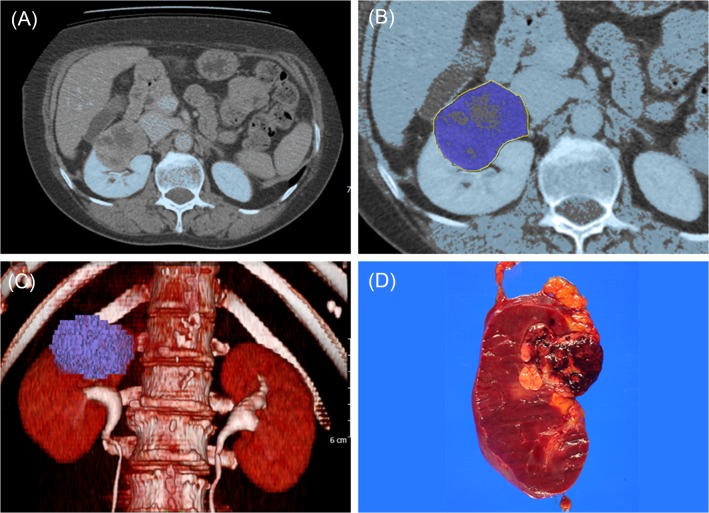
Measurement of tumor volume and gross specimen. (A) Tumor in the right kidney on CT scan. (B) Delineation between tumor and surrounding normal parenchyma (dark blue). (C) 3D tumor volume (dark blue). (D) Tumor in the right kidney (dark brown).

Previously, we reported the measurement of kidney volume in normal individuals and patients with RCC using a segmentation tool program [12, 13]. Cross-sectional CT images of a preoperative venous phase were exported to Xelis software (Infinitt, Seoul, South Korea). First, a threshold of greater than 50 HU was chosen. Second, after the observer had manually rendered the tumor area, the software automatically calculated 3D tumor volume (Fig. 1). RTV was independently measured by two urologists who were blinded to patient information. PTV was measured using an ellipsoid formula (π/6 × height × length × width) from the pathologic result. Patients were excluded from the study if any one of three values regarding tumor size were missing (ex, 4.0 × 3.4 cm).

### Assessment

Baseline characteristics of patients such as age, sex, body mass index (BMI), creatinine, Modification of Diet in Renal Disease-Glomerular filtration rate (MDRD-GFR) [14], tumor side, surgery type, histologic subtype, pathologic T stage, RTS and PTS were investigated. RTV and PTV were compared by PTS (< 4 cm, 4–7 cm, 7–10 cm, and > 10 cm) and histologic subtype (clear cell and non-clear cell). The scatter plot and linear regression line of the RTV and PTV were analyzed in all patients and compared by PTS and histologic subtypes. The correlation between the inter-quartile range (IQR) of the RTV and Fuhrman nuclear grade was also investigated.

### Statistical Analysis

Data for continuous variables were expressed as mean ± standard deviation and compared using the Student’s t-test. Data for categorical variables were expressed as the number of times (percentage) and compared using Pearson’s chi-square test. The comparison between tumor size and volume was performed using the paired t-test. Statistical significance was defined as p < 0.05. Correlation analyses were performed using the Pearson’s correlation coefficient. All data analysis was performed with SPSS statistical software (Statistical Product and Services Solutions, version 20.0, Chicago, IL, USA).

## Results

A total of 482 patients were included in this study. The baseline characteristics of the patients are shown in Table 1. The mean age was 54.9 years (range, 3–86 years), and there were 347 men (72.0%) and 135 women (28.0%). Among these subjects, 304 (63.1%) received RN, and 178 (36.9%) underwent PN. Among the different types of surgeries performed, 148 (28.6%) patients underwent open RN, 89 (18.5%) underwent open PN, 83 (17.2%) had a laparoscopic RN, 36 (7.5%) had a laparoscopic PN, 83 (17.2%) had hand-assisted laparoscopic RN, 19 (3.9%) had hand-assisted laparoscopic PN and 34 (7.1%) had robot-assisted laparoscopic PN. The most common histologic subtype was clear cell (83.6%) and the most common pathologic T stage was T1 (72.8%).

**Table 1:** Baseline characteristics.

Feature	Mean ± SD or n (%)
Age (years)	54.9 ± 11.9
Sex	
Male	347 (72.0)
Female	135 (28.0)
BMI (kg/m^2^)	24.7 ± 3.3
Creatinine (mg/ml)	0.95 ± 0.33
MDRD-GFR (ml/min/1.73m^2^)	87.6 ± 25.5
Tumor side	
Right	227 (47.1)
Left	255 (52.9)
Surgery type	
Radical nephrectomy	304 (63.1)
Partial nephrectomy	178 (36.9)
Histology	
Clear cell	403 (83.6)
Papillary	28 (5.8)
Chromophobe	46 (9.5)
Unclassified	5 (1.0)
Pathologic T stage	
T1	351 (72.8)
T2	42 (8.7)
T3	84 (17.4)
T4	5 (1.0)
RTS (cm)	5.00 ± 2.92
PTS (cm)	4.84 ± 2.95

BMI, body mass index; MDRD, Modification of Diet in Renal Disease; GFR, Glomerular filtration rate; RTS, renal tumor size; PTS, pathologic tumor size.

The mean RTS was 5.00 cm (range, 1.0–17.0 cm) and the mean PTS was 4.84 cm (range, 0.9–16.0) (p<0.001). In addition, when the PTS was < 4 cm, the mean RTS (2.7 cm) and mean PTS (2.5 cm) were statistically different (p<0.001). When the PTS was 4–7 cm, the mean RTS (5.3 cm) and mean PTS (5.0 cm) were also statistically different (p<0.001). However, when the PTS was 7–10 cm, the mean RTS (8.0 cm) and mean PTS (8.0 cm) were not statistically different (p = 0.990), and when the PTS was ≥ 10 cm, the mean RTS (11.3 cm) and mean PTS (11.6 cm) were not statistically different either (p = 0.359).

Among all patients, the mean RTV and mean PTV was 79.0 cm^3^ (range, 0.4–723.7) and 76.9 cm^3^ (range, 0.3–748.8 cm^3^), respectively (p = 0.393), demonstrating no significant difference. But, when the PTS was < 4 cm, the mean RTV and mean PTV was 10.9 cm^3^ and 7.1 cm^3^, respectively (p<0.001). Also, when the PTS was 4–7 cm, the mean RTV and mean PTV was 56.0 cm^3^ and 44.7 cm^3^, respectively (p<0.001). However, when the PTS was 7–10 cm or ≥ 10 cm the mean RTV and mean PTV were not statistically different (p = 0.637, p = 0.180, respectively).

Among the patients with clear cell RCC, the mean RTV (79.9 cm^3^) was larger than the mean PTV (74.8 cm^3^) (p = 0.042). When the PTS was < 4 cm, the mean RTV and mean PTV was 11.7 cm^3^ and 7.1 cm^3^, respectively (p<0.001). Also, when the PTS was 4–7 cm, the mean RTV and mean PTV was 59.3 cm^3^ and 45.6 cm^3^, respectively (p<0.001). However, when the PTS was 7–10 cm or ≥ 10 cm the mean RTV and mean PTV were not statistically different (p = 0.881, p = 0.480, respectively). However, in patients with a non-clear cell histologic subtype, the mean RTV (74.5 cm^3^) was smaller than the mean PTV (88.1 cm^3^), but there was no statistically significant difference (p = 0.055) and when analyzed by PTS, there was no statistically significant difference either (p>0.05) (Table 2).

**Table 2:** RTV and PTV by PTS and histologic subtype.

	N	RTV (cm^3^)	PTV (cm^3^)	*P* value
Total	482	79.0 ± 118.4	76.9 ± 128.2	0.393
PTS (cm)				
< 4	222	10.9 ± 9.9	7.1 ± 6.1	< 0.001
4–7	153	56.0 ± 31.4	44.7 ± 24.0	< 0.001
7–10	63	165.4 ± 61.1	169.3 ± 70.6	0.637
≥ 10	44	378.7 ± 137.0	409.3 ± 141.5	0.180
Clear cell	403	79.9 ± 119.9	74.8 ± 127.4	0.042
PTS (cm)				
< 4	190	11.7 ± 10.3	7.1 ± 6.0	< 0.001
4–7	128	59.3 ± 31.5	45.6 ± 23.9	< 0.001
7–10	52	176.1 ± 60.5	174.8 ± 67.3	0.881
≥ 10	33	400.8 ± 140.0	419.6 ± 157.1	0.480
Non-clear cell	79	74.5 ± 111.1	88.1 ± 132.6	0.055
PTS (cm)				
< 4	32	6.5 ± 6.1	6.8 ± 7.0	0.701
4–7	25	39.1 ± 25.5	40.2 ± 24.8	0.734
7–10	11	115.1 ± 33.2	143.5 ± 83.1	0.208
≥ 10	11	312.4 ± 107.6	378.3 ± 75.8	0.156

RTV, renal tumor volume; PTV, pathologic tumor volume; PTS, pathologic tumor size.

**Fig 2:**
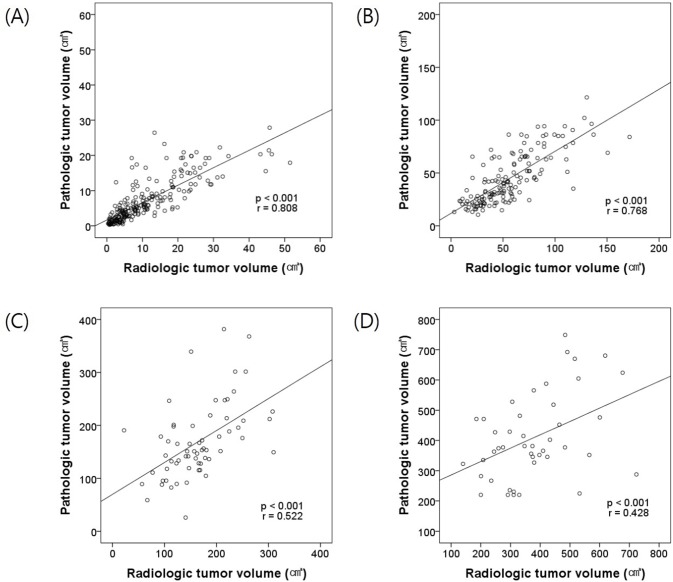
Scatter plots and linear regression lines of RTV and PTV by PTS. (A) PTS < 4 cm, (B) PTS 4–7 cm, (C) PTS 7–10 cm, (D) PTS ≥ 10 cm.

The RTV and PTV had a statistically significant strong positive linear correlation in all patients (p<0.001, r = 0.911). Fig. 2 shows scatter plots and linear regression lines of RTV and PTV by PTS. When the PTS was < 4 cm, RTV and PTV had a statistically significant strong positive linear correlation (p<0.001, r = 0.808), and when the PTS was 4–7 cm, RTV and PTV had a statistically significant strong positive linear correlation (p<0.001, r = 0.768). When the PTS was 7–10 cm, RTV and PTV had a statistically significant moderate positive linear correlation (p<0.001, r = 0.522), and when the PTS was ≥ 10 cm, the RTV and PTV had a statistically significant moderate positive linear correlation (p<0.001, r = 0.428).

When the RTV and PTV were compared according to the tumor pathologic subtype there was a statistically significant strong positive linear correlation (p<0.001, r = 0.918) for clear cell RCC and non-clear cell RCC (p<0.001, r = 0.885).

In the analysis of the Fuhrman nuclear grade according to the IQR of the RTV, when the IQR of the RTV was classified by < first quartile, first—second quartile, second—third quartile and ≥ third quartile, the distribution of Fuhrman grade I was 8 (6.7%), 2 (1.6%), 3 (2.5%) and 1 (0.9%), respectively, the distribution of Fuhrman grade II was 56 (47.1%), 46 (37.7%), 36 (30.3%) and 12 (10.3%), respectively, the distribution of Fuhrman grade III was 51 (42.9%), 69 (56.6%), 72 (60.5%) and 71 (61.2%), respectively, and the distribution of Fuhrman grade IV was 4 (3.4%), 5 (4.1%), 8 (6.7%) and 32 (27.6%), respectively. As the quartile of the RTV increased, the percentage of Fuhrman grade I and II tumors decreased and Fuhrman grade III and IV tumors increased. Thus, as the quartile of the RTV increased, the Fuhrman grade also increased (p<0.001).

## Discussion

This study compared the RTV and PTV in RCC patients according to PTS and histologic subtype, and also investigated the correlation between RTV and Fuhrman nuclear grade. Although there have been several studies comparing the RTS and PTS, there has not yet been an established consensus on the correlation between RTS and PTS. Some studies have reported that the RTS significantly overestimated PTS by 0.1 cm to 0.31 cm [2, 15, 16]. However, in another study, there were no significant difference between RTS and PTS [17–19]. In this study, the mean RTS (5.00 cm) was overestimated by 0.16 cm compared with the mean PTS (4.83 cm), which supports the results of the abovementioned studies. However, tumor size is a one-dimensional measurement and the above studies are limited in their assessment of tumor size due to the variable three-dimensional shape of renal tumors, thus emphasizing the importance of a volumetric method to measure the difference between RTV and PTV as done here.

PTV can be difficult to measure with a high degree of precision. In some studies the PTV was calculated using an ellipsoid formula [7, 10], which is also the method employed in this study. Measurements of renal masses that are not exact ellipsoids and will vary from this approximation to some extent, but to a lesser degree than for a one-dimensional measurement. Similarly, the RTV can also be difficult to accurately measure. Secil et al. calculated the RTV by the conventional method of multiplying three sizes of the tumor on CT images using an ellipsoid formula [9], which suffers from the same limitations mentioned above. They also measured the RTV using a post-processing ViewForum workstation (Philips, Eindhoven, Netherlands) with 3D image processing, including a volume analysis. However, they did not compare these two different volume measuring methods. Aerten et al. measured RTV using a medical imaging post-processing system for volumetric measurements based on MeVisLab software (MeVis Medical Solutions, Bremen, Germany) [8]. RTV was measured in this study from cross-sectional images of a preoperative CT scan using Xelis software (Infinitt, Seoul, South Korea), which can render 3D and calculate RTV easily. This method may offer a more accurate measure of volume regardless of tumor shape. To our knowledge, the current study is the first to show a comparison of the RTV and PTV of renal cell carcinoma according to tumor size and histology subtype.

In this study, the mean RTV and mean PTV were not significantly different, and the RTV and PTV demonstrated a significant strong positive linear correlation in all patients. However, when analyzed according to PTS, the mean RTV was larger than the mean PTV in patients with a PTS < 4 cm and 4–7 cm by 3.8 cm^3^ and 11.3 cm^3^, respectively. A decrease of the PTV in patients with a PTS < 7 cm is explained by tumor shrinkage secondary to vasoconstriction after artery occlusion or blood loss during the operation or after surgery [17]. However, for patients with a PTS ≥7 cm, the RTV and PTV showed a significant positive linear correlation and there was no statistical difference in volume. Large tumor size is associated with tumor necrosis [20], and a significant degree of tumor necrosis is associated with decreased tumor perfusion [21]. Necrosis in large tumors that results in decreased blood perfusion may cause the 3D reconstruction program to exclude those areas from the renal mass measurement taken during venous phase enhancement. Based on the current findings, the calculation of tumor volume was affected by tumor shrinkage after surgery and nonfunctioning necrotic areas. Thus, preoperative RTV is only a reflection of the biologically active portion of the renal mass compared to the postoperative PTV.

When analyzed by histologic subtype, the mean RTV was larger than the mean PTV in the clear cell subtype by 5.1 cm^3^, and when analyzed according to PTS, the mean RTV was larger than the mean PTV in patients with a PTS < 4 cm and 4–7 cm by 4.6 cm^3^ and 13.7 cm^3^, respectively. However, in the non-clear cell subtype, there was no statistical difference. Lee et al. also reported that the mean RTS was significantly larger than the PTS in the clear cell subtype [18]. This could be influenced by the findings that the clear cell subtype shows more enhancement by CT imaging and has increased vascularity compared to other subtypes [22, 23]. Preoperative increased blood supply could result in increased RTV. In addition, a higher degree of tumor shrinkage after the operation occurred in clear cell RCC, which would also increase the difference in mean RTV and PTV in this subtype.

Secil et al. reported that when the renal mass in 46 nephrectomy patients was divided by tumor volume, low-grade tumors (Fuhrman grade I + II) accounted for 69.7% of masses, while high-grade tumors (Fuhrman grade III + IV) accounted for 23.1%, according to a tumor volume ≤ 110 cm^3^. When tumor volume was > 110 cm^3^, 30.3% of tumors were low grade (Fuhrman grade I + II), while 76.9% were high grade (Fuhrman grade III + IV) [9]. The results of this study also revealed that as the RTV quartile increased, the Fuhrman grade increased for all patients.

This study had some limitations. First, this study was retrospective in nature and a single institution analysis, and thus is subject to the bias that is associated with these study types. Second, there was no standard method to measure tumor volume for the different types of programs. Finally, a sub-analysis was not performed for non-clear cell histology due to the relatively small number of cases.

## Conclusions

By using a 3D rendering program, RTV could be measured easily. For a PTS less than 7 cm, the RTV significantly overestimated PTV. In RCCs of the clear cell type, the RTV was significantly larger than the PTV. However, there was a significant positive linear correlation between RTV and PTV. In addition, RTV correlated with pathologic grade. Thus, RTV could be used as an indicator of renal mass aggressiveness preoperatively. Although there were several limitations with the tumor volumetric measuring method, the methods used here and the results will significantly contribute to the study of renal tumor volume.

## References

[1] Alasker A, Williams SK, Ghavamian R. Small renal mass: to treat or not to treat. Current urology reports. 2013;14(1):13–8. 10.1007/s11934-012-0296-3 PubMed . 23192724

[2] Kurta JM, Thompson RH, Kundu S, Kaag M, Manion MT, Herr HW, et al Contemporary imaging of patients with a renal mass: does size on computed tomography equal pathological size? BJU international. 2009;103(1):24–7. 10.1111/j.1464-410X.2008.07941.x PubMed 18710440 PMC2634853

[3] Kathrins M, Caesar S, Mucksavage P, Guzzo T. Renal mass size: concordance between pathology and radiology. Current opinion in urology. 2013;23(5):389–93. 10.1097/MOU.0b013e328363212b PubMed . 23778129

[4] Schlomer B, Figenshau RS, Yan Y, Bhayani SB. How does the radiographic size of a renal mass compare with the pathologic size? Urology. 2006;68(2):292–5. 10.1016/j.urology.2006.03.004 PubMed . 16904439

[5] Herr HW, Lee CT, Sharma S, Hilton S. Radiographic versus pathologic size of renal tumors: implications for partial nephrectomy. Urology. 2001;58(2):157–60 PubMed . 11489688 10.1016/s0090-4295(01)01173-6

[6] Mucksavage P, Kutikov A, Magerfleisch L, Van Arsdalen K, Wein AJ, Ramchandani P, et al Comparison of radiographical imaging modalities for measuring the diameter of renal masses: is there a sizeable difference? BJU international. 2011;108(8 Pt 2):E232–6. 10.1111/j.1464-410X.2010.09977.x PubMed . 21348913

[7] Thiel DD, Jorns J, Lohse CM, Cheville JC, Thompson RH, Parker AS. Maximum tumor diameter is not an accurate predictor of renal cell carcinoma tumor volume. Scandinavian journal of urology. 2013;47(6):472–5. 10.3109/21681805.2013.814071 PubMed . 23883351

[8] Aertsen M, De Keyzer F, Van Poppel H, Joniau S, De Wever L, Lerut E, et al Tumour-related imaging parameters predicting the percentage of preserved normal renal parenchyma following nephron sparing surgery: a retrospective study. European radiology. 2013;23(1):280–6. 10.1007/s00330-012-2582-3 PubMed . 22797982

[9] Secil M, Cullu N, Aslan G, Mungan U, Uysal F, Tuna B, et al The effect of tumor volume on survival in patients with renal cell carcinoma. Diagnostic and interventional radiology. 2012;18(5):480–7. 10.4261/1305-3825.DIR.5346-11.1 PubMed . 22618630

[10] Jorns J, Thiel DD, Lohse CM, Williams A, Arnold ML, Cheville JC, et al Three-dimensional tumour volume and cancer-specific survival for patients undergoing nephrectomy to treat pT1 clear-cell renal cell carcinoma. BJU international. 2012;110(7):956–60. 10.1111/j.1464-410X.2012.10937.x PubMed . 22300498

[11] Choi YA, Kim CK, Park BK, Kim B. Evaluation of adrenal metastases from renal cell carcinoma and hepatocellular carcinoma: use of delayed contrast-enhanced CT. Radiology. 2013;266(2):514–20. 10.1148/radiol.12120110 PubMed . 23151828

[12] Gong IH, Hwang J, Choi DK, Lee SR, Hong YK, Hong JY, et al Relationship among total kidney volume, renal function and age. The Journal of urology. 2012;187(1):344–9. 10.1016/j.juro.2011.09.005 PubMed . 22099987

[13] Jeon HG, Gong IH, Hwang JH, Choi DK, Lee SR, Park DS Prognostic significance of preoperative kidney volume for predicting renal function in renal cell carcinoma patients receiving a radical or partial nephrectomy. BJU international. 2012;109(10):1468–73. 10.1111/j.1464-410X.2011.10531.x PubMed . 21883863

[14] Levey AS, Coresh J, Greene T, Marsh J, Stevens LA, Kusek JW, et al Expressing the Modification of Diet in Renal Disease Study equation for estimating glomerular filtration rate with standardized serum creatinine values. Clinical chemistry. 2007;53(4):766–72. 10.1373/clinchem.2006.077180 PubMed . 17332152

[15] Jeffery NN, Douek N, Guo DY, Patel MI. Discrepancy between radiological and pathological size of renal masses. BMC urology. 2011;11:2 10.1186/1471-2490-11-2 PubMed 21342488 PMC3056852

[16] Choi JY, Kim BS, Kim TH, Yoo ES, Kwon TG. Correlation between Radiologic and Pathologic Tumor Size in Localized Renal Cell Carcinoma. Korean journal of urology. 2010;51(3):161–4. 10.4111/kju.2010.51.3.161 PubMed 20414390 PMC2855455

[17] Alicioglu B, Kaplan M, Yurut-Caloglu V, Usta U, Levent S. Radiographic size versus surgical size of renal masses: which is the true size of the tumor? Journal of BUON: official journal of the Balkan Union of Oncology. 2009;14(2):235–8 PubMed . 19650172

[18] Lee SE, Lee WK, Kim DS, Doo SH, Park HZ, Yoon CY, et al Comparison of radiographic and pathologic sizes of renal tumors. World journal of urology. 2010;28(3):263–7. 10.1007/s00345-010-0511-0 PubMed . 20119641

[19] Ates F, Akyol I, Sildiroglu O, Kucukodaci Z, Soydan H, Karademir K, et al P reoperative imaging in renal masses: does size on computed tomography correlate with actual tumor size? International urology and nephrology. 2010;42(4):861–6. 10.1007/s11255-010-9707-x PubMed . 20148365

[20] Lam JS, Shvarts O, Said JW, Pantuck AJ, Seligson DB, Aldridge ME, et al Clinicopathologic and molecular correlations of necrosis in the primary tumor of patients with renal cell carcinoma. Cancer. 2005;103(12):2517–25. 10.1002/cncr.21127 PubMed . 15880379

[21] Reiner CS, Roessle M, Thiesler T, Eberli D, Klotz E, Frauenfelder T, et al Computed tomography perfusion imaging of renal cell carcinoma: systematic comparison with histopathological angiogenic and prognostic markers. Investigative radiology. 2013;48(4):183–91. 10.1097/RLI.0b013e31827c63a3 PubMed . 23328912

[22] Kim JK, Kim TK, Ahn HJ, Kim CS, Kim KR, Cho KS. Differentiation of subtypes of renal cell carcinoma on helical CT scans. AJR American journal of roentgenology. 2002;178(6):1499–506. 10.2214/ajr.178.6.1781499 PubMed . 12034628

[23] Zhang J, Lefkowitz RA, Ishill NM, Wang L, Moskowitz CS, Russo P, et al Solid renal cortical tumors: differentiation with CT. Radiology. 2007;244(2):494–504. 10.1148/radiol.2442060927 PubMed . 17641370

